# Cross-Sectional Study to Map Nutritional Quality of Meat, Fish, and Dairy Alternatives in Dutch Supermarkets According to the Dutch Food-Based Dietary Guidelines and Nutri-Score

**DOI:** 10.3390/foods12091738

**Published:** 2023-04-22

**Authors:** Sylvie Huybers, Annet J. C. Roodenburg

**Affiliations:** HAS Green Academy, Spoorstraat 62, 5911 KJ Venlo, The Netherlands; a.roodenburg@has.nl

**Keywords:** plant-based, health, nutritional quality, nutri-score, meat alternatives, dairy alternatives, vegetarian diet

## Abstract

Due to a growing challenge to feed the world’s population and an increased awareness to minimize the impact of our food choices on climate change, a more plant-based diet has gained popularity with a growing number of plant-based products on the market. To stimulate a plant-based diet that also improves long-term health, data are needed to monitor whether these products are healthy alternatives to animal-based foods. Therefore, this study inventoried 916 plant-based meat, fish, and dairy alternatives from eight Dutch supermarkets. The nutritional quality of each product was assessed by (1) the Dutch food-based dietary guidelines and (2) the Nutri-Score. The results show that over 70% of meat, fish, and dairy alternatives have an A/B Nutri-Score (indicating high nutritional quality), but do not comply with the Dutch dietary guidelines. This is mainly due to high salt and low vitamin B12 and iron content (meat and fish alternatives) or low protein and calcium levels (dairy alternatives). In conclusion, the majority of plant-based products are nutritionally not full alternatives of the animal-based equivalents; however, there are still opportunities for reformulation. To aid the consumer in making healthy plant-based food choices, a better alignment between the Nutri-Score and the recommended dietary guidelines is needed.

## 1. Introduction

Due to a growing challenge to feed the world’s population and an increased awareness to minimize the impact of our food choices on climate, a more plant-based and less animal-based diet has gained popularity. This is illustrated in a study including 10 European countries, where plant-based eaters represent 7% of the population, whereas 30% followed a flexitarian diet [1]. A flexitarian was defined as a person who sometimes eats meat but is trying to reduce meat consumption and often chooses plant-based alternatives instead. This expanding group of people who deliberately choose to eat no or less meat resulted in an increase of 49% in European sales value of plant-based food, mainly meat and dairy alternatives, in the period 2018–2020 [2]. Also, foodservice sales of meat and dairy alternatives have increased by 145% in The Netherlands over the last five years [3]. This shows the increasing importance of plant-based products in our diet.

Besides being a sustainable diet [4], the health benefits of a plant-based diet have also received increased attention. A more plant-based diet can reduce the risk of cardiovascular diseases compared to an animal-based diet [5,6,7,8]. For flexitarians, the most important factors when choosing a plant-based food product are taste and health [1]. Plant-based food alternatives should therefore be healthy products that deliver the required amount of nutrients and fit into a healthy diet based on dietary guidelines. In meat alternatives, low protein and iron and high salt content have been described [9,10,11,12], whereas in dairy alternatives, a low protein and calcium content have been reported [13,14,15,16,17]. This could lead to deficiencies, especially when the diet turns into a more complete vegetarian diet.

More insight into the nutritional value of meat, fish, and dairy alternatives is needed to map nutritional concerns related to possible health effects on the long term. It enables the development of products that better fit into a healthy plant-based diet by defining recommendations for nutritional (re)formulation. Therefore, the aim of the current study was to investigate the nutritional quality of the most consumed vegetarian meat, fish, and dairy alternatives on the Dutch market. To evaluate the nutritional quality of the products, two different methods were used: (1) the Dutch food-based dietary guidelines and (2) the Nutri-Score.

## 2. Materials and Methods

### 2.1. Product Selection and Data Collection

A total of 916 meat, fish, and dairy alternatives were selected from eight Dutch supermarkets in the period of March–May 2021. Products were found using search terms (vegetarian, plant-based, meat and/or fish alternative, milk and/or cheese alternative, protein drink) in the webshop of the supermarket. All available products that fell into one of the two categories—(1) ready-made meat, fish, and cold cuts alternatives, (2) plant-based protein drinks, desserts, and cheeses—were selected. Cold cuts were defined as products that mimic the structure and taste of traditional lunch meats, like salami or bacon. Fish alternatives are mainly products that mimic fish burgers or fish fingers. The division and nomenclature of the (sub)categories were in alignment with the Kies-ik-gezond app of the Dutch Nutrition Centre. Information about nutritional value (energy, protein, total sugar, saturated fatty acid, salt, calcium, iron, and vitamin B12 as expressed per 100 g) and product ingredients were obtained from the nutritional table and ingredient list as mentioned in the webshop. Online product information was not available for two supermarkets. Products (N = 42) that were not included in the product database after the prior online selection were added via a physical visit to these two supermarkets. Photos were taken of both the front and back of the package from which the nutritional value and product ingredients were collected. In this way, necessary nutritional information was obtained for all selected products without missing data. Protein energy % (E%) was calculated as the amount of protein per 100 g divided by total energy from protein (i.e., protein per 100 g × 4 Kcal) × 100. In case a nutrient was not mentioned in the nutritional table, the available amount in the product was assumed to be zero.

### 2.2. Evaluation of Nutritional Quality

#### 2.2.1. Dutch Food-Based Dietary Guidelines (the Wheel of Five)

The Dutch food-based dietary guidelines are presented in the Wheel of Five, which consists of five main product categories (i.e., Vegetables and fruits, Bread and grain/cereal products and potatoes, Dairy and nuts and fish and legumes and meat and eggs, Drinks, Spreading and cooking fats), further divided into subcategories. Each category consists of products that meet the criteria for a healthy diet with recommendations for amounts of daily intake [18]. The selected food products in the inventory were scored according to the criteria of the subcategories ready-made vegetarian meat alternatives, plant-based protein drinks and desserts, and plant-based cheese alternatives (Table 1). Vitamin B1 and B12 are interchangeable in the criteria. Since vitamin B12 is mainly used as fortification in plant-based meat alternatives, this study focused only on vitamin B12 content. Cold cuts alternatives were separately analyzed from the ready-made vegetarian meat alternatives since the product properties between these subgroups vary considerably. Examples of these cold cuts alternatives are plant-based pate, vegetarian chicken curry spread, or slices of plant-based bacon or sausage. No criteria were available for cold cuts and fish alternatives. Therefore, the criteria for ready-made vegetarian meat alternatives were used. A product was included in the Wheel of Five and, as such, classified as healthy when all criteria were met [19]. For each product group, the ratio between the mean value of a nutrient and the corresponding criteria level was calculated.

#### 2.2.2. Nutri-Score

**Figure 1:**
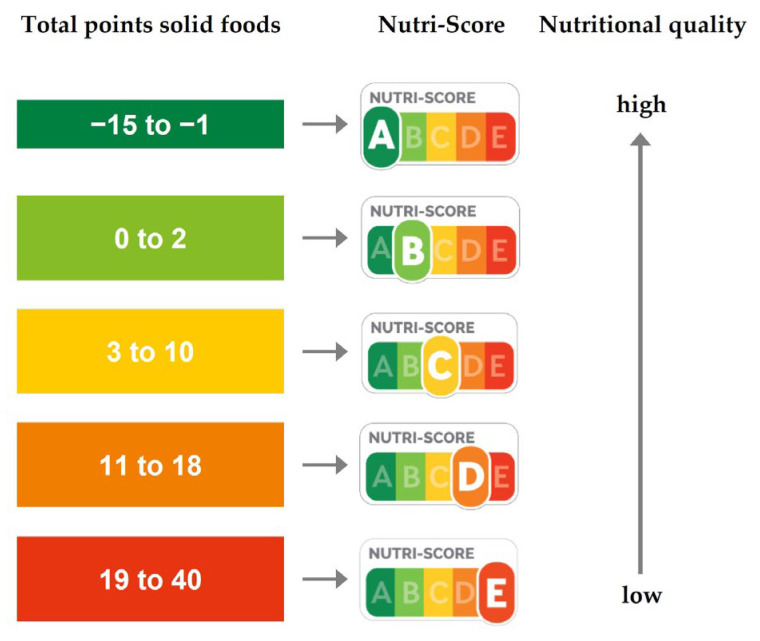
The Nutri-Score front-of-pack logo. Total points are based on the sum of points for nutrients with a positive effect on health (fiber, protein content, % fruit and vegetables) and nutrients with a negative effect on health (energy density, sugars, fatty acids, sodium). The score ranges from products with a high nutritional quality (A, dark green) to products with a low nutritional quality (E, red).

The Nutri-Score of the products was calculated according to the guidelines of Santé publique France [20]. The Nutri-Score features five categories of nutrient quality by combining letters (A to E) and colors (green to red). It ranges from a dark green A score (associated with the highest nutritional quality) to a red E score (associated with the lowest nutritional quality) (Figure 1). The Nutri-Score algorithm assigns points based on nutrient content in 100 g of solid food or cheese or 100 mL of beverage. Positive points (between 0 to 5) are obtained for each nutrient that has a good impact on health, i.e., the percentage of fruits, vegetables, pulses, nuts, and rapeseed, walnut and olive oils, and the amount of fiber and protein. The amount of fruits, vegetables, pulses, nuts, and rapeseed, walnut and olive oils was calculated by the sum of each individual component (expressed as % in the ingredient list). Negative points (between 0 to 10) are attributed for each nutrient that negatively affects health, i.e., energy density and the amount of sugars, saturated fatty acids, and sodium. Total points (between −15 and +40) are calculated by subtracting the sum of positive points from the sum of negative points. The final Nutri-Score is based on the cutoff points as indicated in Figure 1. For ready-made meat and fish alternatives and plant-based protein drinks and desserts, the Nutri-Score algorithm for solid foods was used according to the Nutri-Score guidelines. For plant-based cheese alternatives, the Nutri-Score algorithm for cheeses was used. The Nutri-Score was based on the individual calculations and not on the Nutri-Score presented on the package, if applicable.

### 2.3. Data Analysis

Data were collected in Excel (Microsoft) and analyzed using SPSS statistical software (IBM). Normal distribution of the nutritional values was evaluated with the Shapiro–Wilk test. All values followed a not-normal distribution. Therefore, values are expressed as median (25–75 percentile). To evaluate whether the nutritional values are different between the product groups or between the Nutri-Score categories, the Kruskal–Wallis non-parametric test for k-independent samples was used. Statistical significance was set at *p* < 0.05.

## 3. Results

### 3.1. Nutritional Quality of Meat, Fish, and Cold Cuts Alternatives

In total, 445 meat alternatives, 16 fish alternatives, and 59 cold cuts alternatives were compared with criteria of the Dutch food-based dietary guidelines [18] (Table 2). In meat alternatives, salt content meets the criteria level in 39% of products, while fatty acid and protein content are in line with the criteria in more than 72% of the products. Protein content is significantly lower in fish and cold cuts alternatives compared to meat alternatives, with 44% and 25% of products that meet the criteria, respectively. Meat, fish, and cold cuts alternatives are all low in iron (30%, 25%, 8%) and vitamin B12 content (28%, 25%, 5%). Cold cuts alternatives have lower nutritional value compared to ready-made meat alternatives for all analyzed nutrients. Overall, just 3%, 13%, and 0% of meat, fish, and cold cuts alternatives, respectively, meet all the criteria of the Dutch food-based dietary guidelines.

**Figure 2:**
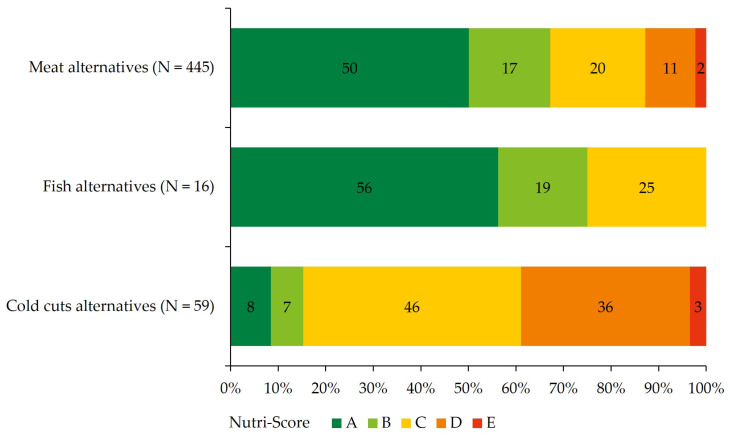
Nutri-Score distribution of meat, fish, and cold cuts alternatives. The Nutri-Score ranges from the product with the highest nutritional quality (dark green A) to products with the lowest nutritional quality (red E).

Beside the criteria of the Dutch food-based dietary guidelines, the nutritional quality of the products is expressed as the Nutri-Score (Figure 2). Meat alternatives (67%) and fish alternatives (75%) are classified in the highest Nutri-Score levels A and B, indicating a high nutritional quality. Of the cold cuts alternatives, 85% falls into the Nutri-Score levels C, D, and E claiming to be products with medium-to-low nutritional quality.

**Figure 3:**
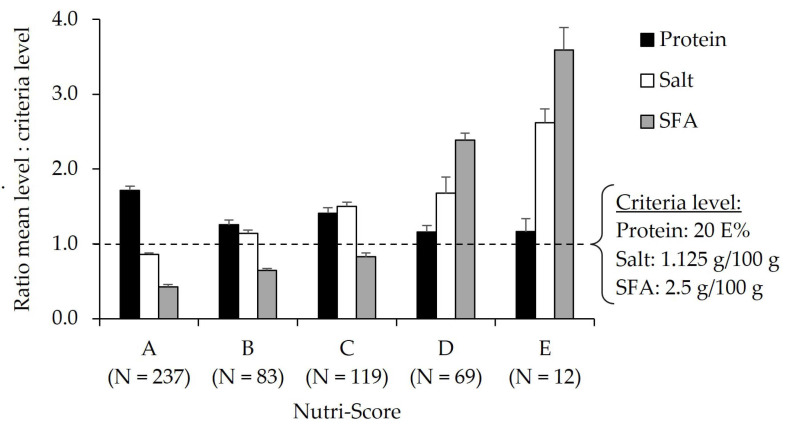
Ratio between mean levels of protein, salt, and saturated fatty acids (SFA) and the corresponding criteria levels of the Dutch food-based dietary guidelines in meat and fish alternatives (N = 520) per Nutri-Score category. A ratio >1 indicates mean levels above the criteria level, and a ratio <1 indicates mean levels below the criteria level. The Nutri-Score ranges from the product with the highest nutritional quality (A) to products with the lowest nutritional quality (E).

Figure 3 shows the ratio between mean protein, salt, and saturated fatty acid content and the corresponding criteria levels of the Dutch food-based dietary guidelines for the total group of meat and fish alternatives (N = 520). A ratio >1 indicates mean levels above the criteria level, and a ratio <1 indicates mean levels below the criteria level. Data are stratified according to the Nutri-Score. It shows that products with Nutri-Score levels D and E contain significantly higher amounts of saturated fatty acids compared to products with a Nutri-Score at the A, B, and C levels (*p* < 0.001). In addition, salt content gradually increases from Nutri-Score A to E (*p* < 0.001). Protein content is comparable between Nutri-Score levels except for Nutri-Score A, where products have a significantly higher protein content compared to all the other levels (*p* < 0.001).

### 3.2. Nutritional Quality of Milk, Dessert, and Cheese Alternatives

In total, 211 milk alternatives, 127 dessert alternatives, and 58 cheese alternatives were analyzed for nutritional quality (Table 3). Milk and cheese alternatives meet the protein criteria level in 20% and 2%, respectively. Protein content was significantly higher in dessert alternatives, although 71% did not reach the criteria levels. Median levels are zero for both calcium and vitamin B12 content in all product categories. However, for milk and dessert alternatives, 36% and 31% of products meet the criteria levels for calcium and 31% and 28% for vitamin B12. Cheese alternatives have a significantly higher level of saturated fatty acids and total sugars compared to milk and dessert alternatives. In total, 9% of milk alternatives, 3% of dessert alternatives, and 0% of cheese alternatives meet all the criteria.

**Figure 4:**
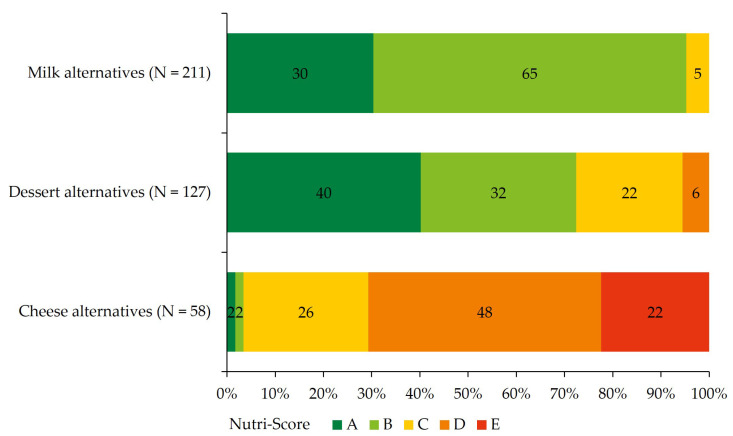
Nutri-Score distribution of milk, dessert, and cheese alternatives. The Nutri-Score ranges from the product with the highest nutritional quality (dark green A) to products with the lowest nutritional quality (red E).

Beside the criteria of the Dutch food-based dietary guidelines, the nutritional quality of the products is expressed as the Nutri-Score (Figure 4). The majority (95%) of milk alternatives have a Nutri-Score level of A or B and are defined as products with high nutritional quality. The amount of dessert alternatives with an A or B score is 72%. Cheese alternatives have low nutritional quality with Nutri-Score D and E in 70% of the products.

**Figure 5:**
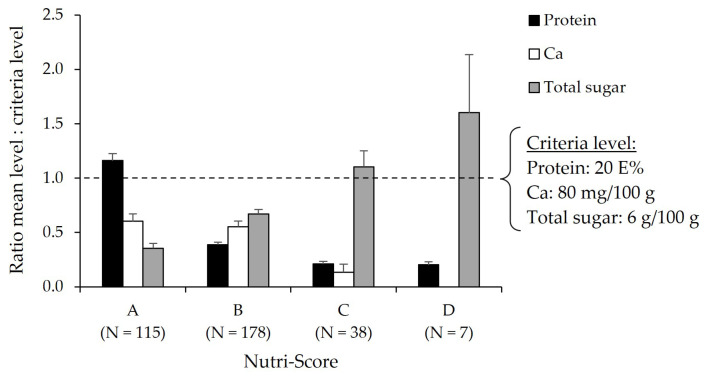
Ratio between mean levels of protein, calcium, and total sugar and the corresponding criteria levels of the Dutch food-based dietary guidelines in milk and dessert alternatives (N = 338) per Nutri-Score category. A ratio >1 indicates mean levels above the criteria level, and a ratio <1 indicates mean levels below the criteria level. The Nutri-Score ranges from the product with the highest nutritional quality (A) to products with the lowest nutritional quality (E).

Figure 5 shows the whole group of plant-based protein drinks and desserts (N = 338) divided by Nutri-Score level, in which the ratio between the mean protein, calcium, and total sugar levels and the corresponding Dutch food-based dietary guidelines are expressed. The milk and dessert alternatives with Nutri-Score level A have a higher protein content compared to the other Nutri-Score levels (*p* < 0.001). Also, total sugar content in products with Nutri-Score level A is significantly more in alignment with the criteria compared to the other Nutri-Score categories (*p* < 0.001). For calcium, products with Nutri-Score level A or B have a more favorable ratio compared to levels C and D (*p* < 0.001).

## 4. Discussion

This study shows that the majority of meat, fish, and dairy alternatives in Dutch supermarkets do not meet the requirements of the Dutch food-based dietary guidelines and are, therefore, not recommended as part of a healthy diet. The Nutri-Score shows in general a more positive view on the nutrient quality of meat, fish, and dairy products.

Various studies investigated the nutritional profile of meat alternatives and compared it to the original meat products using criteria of the front-of-pack logos such as Nutri-Score [9,10,11,12]. Overall, these studies show that meat alternatives have lower amounts of energy and saturated fatty acids and higher amounts of fiber, carbohydrates, and sugar. Protein and salt content are roughly comparable, but varied based on meat type and/or protein source. The Nutri-Score is generally more favorable for plant-based meat alternatives. This study did not include animal-based products, but focused on the nutritional quality comparing the criteria of the Dutch food-based dietary guidelines and the Nutri-Score. Meat alternatives were mainly classified in the Nutri-Score categories A and B, indicating high nutritional quality. In literature, similar Nutri-Score levels were found only for the categories of plant-based steak, burger, or fillet [9,10,11]. This suggests that the selection of the type of meat alternatives influences the results. In contrast to the Nutri-Score outcomes, we found that meat alternatives had low nutritional quality when taking into account the criteria of the Dutch food-based dietary guidelines. This is mainly caused by high amounts of salt and low amounts of iron and vitamin B12, as compared to the criteria cutoff levels. A possible explanation for the discrepancy with the Nutri-Score is that iron and vitamin B12 are not taken into account in the Nutri-Score. Furthermore, negative points from one nutrient, for example salt, can be compensated for by the positive points from another nutrient in the Nutri-Score algorithm.

Cold cuts alternatives were analyzed as a separate subcategory. The animal-based cold cuts mainly consist of processed (red) meat and are high in saturated fatty acids and salt [11,12]. Due to these negative health aspects, they are not included in the Wheel of Five of the Dutch dietary guidelines. In this study, also cold cuts alternatives were high in saturated fatty acids and salt, which is in line with the literature [11,12]. Nutritional quality seems to be of less importance in this product category. Nonetheless, sodium reduction could be an interesting opportunity for product development, because salt from cold cuts alternatives, as well as from ready-made meat alternatives, negatively contributes to the already high sodium consumption in The Netherlands [21].

Since more people are replacing traditional meat products with meat alternatives, specifically iron and vitamin B12 content are of importance. Meat contributes to haem iron and vitamin B12 intake in the Dutch population for, respectively, 89% and 30% [21]. Interestingly, one third of Dutch women showed lower iron intake than the recommended average daily intake [22]. Anemia due to iron deficiency is still prevalent in 12.3 per 1000 people in The Netherlands [23]. Vitamin B12 deficiency is currently not frequently present, but can become relevant in a vegan diet where animal-based products containing high vitamin B12 levels are omitted. Fortification can therefore be essential to obtain the recommended dietary allowance, especially for iron which is present in the highly absorbable haem iron form in meat in contrast to the less absorbable iron in meat alternatives [24]. Iron and vitamin B12 fortification is an important point to address for product reformulation and should be recommended for all meat alternatives, including fish and cold cuts alternatives.

Concerning plant-based protein drinks and desserts, this study found amounts of protein and calcium content that are below the criteria levels of the Dutch food-based dietary guidelines. This is in line with previous studies that found low levels of calcium and protein compared to animal-based milk products [10,13,15,25]. Like with meat alternatives, the criteria of the Dutch food-based dietary guidelines and the Nutri-Score are not in alignment for dairy alternatives. The current study showed that almost all milk alternatives score high on nutritional quality with a Nutri-Score level A or B, but do not meet the requirements for protein and calcium content. For milk and dessert alternatives, many protein sources with poor protein quality are used, like rice, almonds, and coconut. The protein content of plant-based sources is not only lower compared to animal-based sources, but also has a less balanced essential amino acid profile and lower ileal digestibility [26]. Rating the nutritional value of dairy alternatives should therefore emphasize more on protein quality and not exclusively on protein content. Milk and dessert alternatives had either calcium fortified to the recommended calcium levels or did not contain any calcium. Protein source and calcium fortification were highly dependent on the specific brand. Cheese alternatives had neglectable protein content and were not fortified with calcium. In the Dutch population, 24% and nearly 60% of, respectively, protein and calcium intake are acquired from dairy products [21]. Since it is well established that calcium from plant-based sources has lower bioavailability compared to animal-based sources [27], product fortification or the use of calcium supplements is relevant in the case of a predominantly plant-based diet. It lowers the risk of developing weaker bones [6]. Therefore, calcium fortification is recommended in all dairy alternatives, including cheeses.

The differences in nutritional values between animal-based products and their plant-based alternatives can make it difficult for the consumer to judge the nutritional quality of a meat or dairy alternative. To inform the consumers about the product quality and aid in making healthy choices, different front-of-pack logos have been introduced such as the Nutri-Score. The Nutri-Score is planned to be realized in The Netherlands in 2023, but it is under discussion since it is not in line with all the criteria of the food-based dietary guidelines or the Wheel of Five for several product groups [28]. This is consistent with the results from this study for the product category meat and dairy alternatives. A high Nutri-Score on plant-based alternatives can be misleading for the consumer, because the products are not a nutritionally complete substitute and are therefore not included in the Wheel of Five and recommended as part of a healthy diet. The Dutch government is waiting for an improved algorithm to bring the Nutri-Score more in line with the Dutch dietary guidelines. For example, in the current algorithm, the Nutri-Score of plant-based protein drinks is calculated within the general/solid foods category. A scientific committee is considering the inclusion of plant-based protein drinks in the beverages category, which has more strict criteria on protein content and would generate Nutri-Scores that are more in line with the Dutch food-based dietary guidelines [29,30]. Including iron, vitamin B12, calcium, and protein quality in the Nutri-Score criteria could make the Nutri-Score more future-proof for the growing plant-based market. We believe this is necessary if we want to achieve that the Nutri-Score actually leads to more healthy food choices. This is supported by a recent study concluding that, with the current algorithm, there is not enough scientific evidence to substantiate a cause-and-effect relationship between the Nutri-Score and more healthy food choices in the supermarket [31]. Another option is to make a separate Nutri-Score algorithm for plant-based alternatives.

This research gained more insight into the nutritional quality of meat and dairy alternatives, but also has some limitations. It did not take into account all nutrients that could influence the nutritional quality of a product. For example, plant-based products have a higher fiber content compared to animal-based products [9,10,32]. It is widely established that fiber intake can positively affect health [33]. Furthermore, meat and dairy alternatives are more ultraprocessed compared to most animal-based equivalents. Ultraprocessed food is assumed to increase energy intake, possibly via changes in food structure and eating rate [34,35]. Furthermore, there are indications that food processing could also influence digestibility or absorption of nutrients [36]. More research is necessary to evaluate the effect of highly processed milk and dairy alternatives on long-term health. Finally, this study includes a selected group of products available from a limited number of Dutch supermarkets. A more extensive study including all available products also from other European countries and using front-of-pack criteria that are applied in other countries (Keyhole, Choices Programme) could be an opportunity to make the conclusions more representative for a broader market.

Overall, we conclude that meat and dairy alternatives in Dutch supermarkets do not fully align with the criteria as listed in the Dutch food-based dietary guidelines. There are still opportunities for reformulation to come closer to the nutritional values of the animal-based products without affecting taste, such as the addition of iron, vitamin B12, and calcium, and the use of plant-based protein sources with the highest protein quality. A Nutri-Score that takes these nutritional aspects along in a new algorithm for plant-based alternatives would increase its reliability. This will aid consumers in making healthy plant-based choices. Besides, it would stimulate the food companies to improve the nutritional quality of their products. This becomes increasingly relevant when people change towards a more plant-based diet and especially when more people choose a vegetarian or vegan diet.

## Figures and Tables

**Table 1:** Criteria for ready-made vegetarian meat alternatives, plant-based protein drinks and desserts, and plant-based cheese alternatives according to the Dutch food-based dietary guidelines.

Nutrient	Unit	Ready-Made Vegetarian Meat Alternatives *^1^	Plant-Based Protein Drinks and Desserts	Plant-Based Cheese Alternatives
Saturated fatty acids	g/100 g	≤2.5	≤1.1	≤14
Trans fatty acids	g/100 g	≤0.1	Not added	Not added
Total sugar	g/100 g	Not added	≤6	Not added
Protein	E%	≥20	≥20	≥20
Salt *^2^	g/100 g	≤1.125	≤0.15	≤2.05
Calcium	mg/100 g	NA	≥80	≥500
Iron	mg/100 g	≥0.8	NA	NA
Vitamin B12	mcg/100 g	≥0.24	≥0.24	≥0.24
Vitamin B1 *^3^	mcg/100 g	≥0.06	NA	NA

*^1^ criteria also used for cold cuts alternatives and vegetarian fish alternatives; *^2^ 1.125 g salt = 450 mg Na, 0.15 g salt = 60 mg Na, 2.05 g salt = 820 mg; *^3^ = vitamin B12 and/or vitamin B1, NA = not applicable. E% = percentage protein energy content was calculated as the amount of energy from proteins (kcal/100 g) divided by total energy (kcal/100g) × 100% using the conversion factor 4 kcal/gram protein. Products that meet all criteria are included in the Wheel of Five and indicated as healthy [18].

**Table 2:** Nutritional quality of meat, fish, and cold cuts alternatives compared to the Dutch food-based dietary guidelines.

Nutrient	Unit	Meat Alternatives (N = 445)		Fish Alternatives (N = 16)		Cold Cuts Alternatives (N = 59)	
Saturated fatty acids	g/100 g	1.20	(0.80–2.40) ^a^	0.90	(0.60–1.70) ^a^	1.50	(1.40–3.40) ^b^
	Meet criteria (%)	77		88		68	
Salt	g/100 g	1.30	(1.00–1.53) ^a^	1.06	(1.00–1.31) ^a^	1.70	(1.40–1.90) ^b^
	Meet criteria (%)	39		63		12	
Protein	E%	28.96	(18.56–42.34) ^a^	15.20	(9.63–25.10) ^b^	13.16	(8.16–20.00) ^b^
	Meet criteria (%)	72		44		25	
Iron	mg/100 g	0.00	(0.00–2.10) ^a^	0.00	(0.00–1.58) ^a, b^	0.00	(0.00–0.00) ^b^
	Meet criteria (%)	30		25		8	
Vitamin B12	µg/100 g	0.00	(0.00–0.30) ^a^	0.00	(0.00–0.29) ^a, b^	0.00	(0.00–0.00) ^b^
	Meet criteria (%)	28		25		5	
Meet all criteria	%	3		13		0	

Data are expressed as median (25–75 percentile) and amount of products that meet criteria (%). Different letters (^a, b^) within the same row indicate a significant difference (*p* < 0.05) between the product categories. E% = energy %.

**Table 3:** Nutritional quality of milk, dessert, and cheese alternatives compared to the Dutch food-based dietary guidelines.

Nutrient	Unit	Milk Alternatives (N = 211)		Dessert Alternatives (N = 127)		Cheese Alternatives (N = 58)	
Saturated fatty acids	g/100 g	0.30	(0.10–0.40) ^a^	0.50	(0.30–2.40) ^b^	11.30	(6.00–20.00) ^c^
	Meet criteria (%)	92		68		57	
Total sugar	g/100 g	2.50	(0.20–4.80) ^a^	4.10	(0.70–9.07) ^b^	1.30	(0.00–2.55) ^c^
	Meet criteria (%)	86		58		100	
Protein	E%	6.78	(2.55–15.38) ^a^	9.89	(4.05–21.14) ^b^	5.96	(1.90–13.99) ^a^
	Meet criteria (%)	20		29		2	
Salt	g/100 g	0.10	(0.08–0.13) ^a^	0.10	(0.05–0.18) ^a^	1.44	(1.00–1.93) ^b^
	Meet criteria (%)	92		72		84	
Calcium	mg/100 g	0.00	(0.00–120) ^a^	0.00	(0.00–120) ^a^	0.00	(0.00–0.00) ^b^
	Meet criteria (%)	36		31		0	
Vitamin B12	µg/100 g	0.00	(0.00–0.38) ^a^	0.00	(0.00–0.38) ^a, b^	0.00	(0.00–0.00) ^b^
	Meet criteria (%)	31		28		7	
Meet all criteria	%	9		3		0	

Data are expressed as median (25–75 percentile) and amount of products that meet criteria (%). Different letters (^a, b, c^) within the same row indicate a significant difference (*p* < 0.05) between the product categories. E% = energy %.

## Data Availability

The datasets generated for this study are available on request to the corresponding author.
